# Acetazolamide effects on natriuresis and diuresis in acute heart failure treated with furosemide and SGLT2i (SANDI)

**DOI:** 10.1093/eschf/xvag096

**Published:** 2026-04-01

**Authors:** Julia González González, Juan Górriz Magaña, Pau Llàcer, Aleix Fort Pal, Joan Carles Trullàs, Isabel Zegrí-Reiriz, Pablo Díez-Villanueva, Alberto Esteban Fernandez, Laura Pertejo Manzano, Ana Belén Méndez Fernández, Pedro Caravaca Pérez, Juan Carlos López-Azor, Ricardo Martínez, Marianna D’Amato, Concepción Fernández Pascual, Eva Ruiz-Ruiz, Esteban Pérez Pisón, María Berenguel Anter, Marta Cobo Marcos

**Affiliations:** Department of Cardiology, Hospital Universitario Puerta de Hierro, C/Joaquin Rodrigo 2, Majadahonda, 28222 Madrid, Spain; Department of Cardiology, Hospital Universitario Central de la Defensa Gómez Ulla, Madrid, Spain; Department of Internal Medicine, Hospital Universitario Ramón y Cajal, IRYCIS, Madrid, Spain; Department of Medicine and Medical Specialties, Facultad de Medicina y Ciencias de la Salud, Universidad de Alcalá, Madrid, Spain; Department of Cardiology, Hospital Universitario de Girona Doctor Josep Trueta, Girona, Spain; Department of Internal Medicine, Hospital D’Olot I Comarcal de la Garrotxa, Olot, Girona, Spain; Tissue Repair and Regeneration Laboratory (TR2Lab), Institute for Research and Innovation in Life and Health Sciences in Central Catalonia (IrisCC), Faculty of Medicine, University of Vic—Central University of Catalonia (UVic-UCC), Vic, Barcelona, Spain; Department of Cardiology, Hospital de la Santa Creu I Sant Pau, Barcelona, Spain; Department of Cardiology, Hospital Universitario de la Princesa, Madrid, Spain; Department of Cardiology, Hospital Universitario Severo Ochoa, Madrid, Spain; Department of Cardiology, Hospital Universitario Virgen de las Nieves, Granada, Spain; Department of Cardiology, Hospital Universitario Vall D'Hebron, Barcelona, Spain; Department of Cardiology, Hospital Clinic de Barcelona, Barcelona, Spain; Department of Cardiology, Hospital Universitario Puerta de Hierro, C/Joaquin Rodrigo 2, Majadahonda, 28222 Madrid, Spain; Department of Cardiology, Hospital Universitario Puerta de Hierro, C/Joaquin Rodrigo 2, Majadahonda, 28222 Madrid, Spain; Department of Cardiology, Hospital Universitario Central de la Defensa Gómez Ulla, Madrid, Spain; Department of Cardiology, Hospital Universitario Central de la Defensa Gómez Ulla, Madrid, Spain; Department of Internal Medicine, Hospital D’Olot I Comarcal de la Garrotxa, Olot, Girona, Spain; Department of Internal Medicine, Hospital Universitario Ramón y Cajal, IRYCIS, Madrid, Spain; Department of Medicine and Medical Specialties, Facultad de Medicina y Ciencias de la Salud, Universidad de Alcalá, Madrid, Spain; Department of Cardiology, Hospital Universitario de Girona Doctor Josep Trueta, Girona, Spain; Department of Cardiology, Hospital Universitario Puerta de Hierro, C/Joaquin Rodrigo 2, Majadahonda, 28222 Madrid, Spain; Centro de Investigación Biomédica en Red (CIBER Cardiovascular), Madrid, Spain

**Keywords:** Acetazolamide, SGLT2i, Natriuresis, Acute heart failure, Diuretic

## Abstract

**Aims:**

Early adjunctive therapy with intravenous acetazolamide in combination with loop diuretics has been shown to enhance decongestion in acute heart failure (AHF). However, patients receiving sodium–glucose cotransporter-2 inhibitors (SGLT2i) were excluded from previous acetazolamide trials, and the efficacy of this combination remains unknown. This study aims to evaluate the natriuretic and diuretic effects of acetazolamide in patients with AHF treated with intravenous furosemide and concomitant SGLT2i therapy.

**Methods:**

The SANDI study is a prospective, multicentre, observational study enrolling 64 patients hospitalized with AHF and clinical signs of congestion. All participants will receive intravenous loop diuretics and an SGLT2i within the first 24 h of admission. If congestion persists 24 h after SGLT2i initiation, intravenous acetazolamide will be administered up to 2 consecutive days. The primary endpoint is the change in natriuresis within the first 24 h following acetazolamide administration. Secondary endpoints include changes in diuresis, body weight, ADVOR clinical congestion score, and ultrasound-based congestion parameters.

**Conclusion:**

The SANDI study will determine whether adding acetazolamide to intravenous furosemide and SGLT2i enhances early natriuresis and diuresis in patients with AHF and persistent congestion.

## Introduction

Acute heart failure (AHF) remains a leading cause of hospital admission and is associated with substantial morbidity and mortality.^1,2^ Congestion is the hallmark of AHF, and its persistence is associated with greater symptom burden, increased risk of early rehospitalization, and worse clinical outcomes,^3–5^ underscoring the importance of achieving timely and effective decongestion. Although intravenous loop diuretics are the cornerstone of decongestive therapy, a suboptimal diuretic response occurs in approximately 25–30% of patients, particularly among those with advanced disease, renal dysfunction, or prior high-dose diuretic exposure.^3–5^ Proteomic analyses in AHF have reinforced the biological context underlying diuretic response. Notably, proteins linked to diminished diuretic response were also associated with increased mortality risk.^6^

In recent years, early combination diuretic strategies have emerged as a key approach to optimize decongestion in AHF. The CLOROTIC (Combining Loop with Thiazide Diuretics for Decompensated Heart Failure) trial demonstrated that the addition of hydrochlorothiazide enhances decongestion,^7^ while the ADVOR (Acetazolamide in Acute Decompensated Heart Failure with Volume Overload) trial showed that early intravenous acetazolamide significantly improves natriuresis, diuresis, and clinical decongestion compared with standard loop-diuretic therapy alone.^8^ Notably, however, ADVOR excluded patients receiving sodium–glucose cotransporter-2 inhibitors (SGLT2i).

SGLT2i have now become a foundational therapy across the heart failure spectrum, providing cardiorenal protection and promoting osmotic diuresis and variable natriuresis.^9–15^ Similar to acetazolamide, these agents modulate proximal tubular sodium handling through effects on the sodium–hydrogen exchanger (NHE3), albeit via distinct mechanisms.^16–19^ Nevertheless, the combined natriuretic and decongestive effects of these two proximal-acting agents remain unexplored in contemporary clinical practice.

Natriuresis represents a physiological measure of tubular sodium handling and diuretic responsiveness in AHF.^20^ An early natriuretic response has been associated with more efficient decongestion and shorter hospital length of stay.^21^ Moreover, recent studies suggest that early sodium excretion may represent a more clinically meaningful marker of decongestion than urine output alone.^22^ Evidence supporting a consistent natriuretic effect of SGLT2 inhibitors in patients with AHF remains limited.^23^ The ADVOR trial demonstrated that proximal tubular modulation with acetazolamide enhances natriuresis;^18^ however, whether this effect persists in patients receiving concomitant SGLT2 inhibitors remains unknown.

The SANDI study aims to determine whether adjunctive intravenous acetazolamide provides additional natriuretic and diuretic benefits in patients with AHF already treated with loop diuretics and SGLT2i, addressing a key evidence gap in the management of acute decompensation.

## Study design

The SANDI study is a prospective, multicentre, observational study that will be conducted across 11 hospitals in Spain. It will enrol patients hospitalized for AHF presenting with clinical evidence of fluid overload. The study will evaluate natriuresis at 24 and 48 h following intravenous acetazolamide administration in patients with persistent congestion despite treatment with intravenous furosemide and an SGLT2i.

Upon admission, all patients will receive intravenous loop diuretics according to current European guideline recommendations.^24^ For those not previously treated with an SGLT2i, therapy will be initiated within the first 24 h. Congestion will be reassessed 24 h after combined loop diuretic and SGLT2i therapy. In line with the ADVOR trial,^8^ patients with persistent congestion (ADVOR score >1) will receive intravenous acetazolamide (500 mg once daily) for up to two consecutive days. Details of the ADVOR score are provided in *Table 1*. During hospitalization, participants will be encouraged to limit their daily dietary sodium and fluid intake to 3 g and 1500 ml, respectively.

**Table 1:** ADVOR-score

Oedema	No oedema (SCORE 0)	Trace oedema (pitting disappear immediately) (SCORE 1)	Clear pitting oedema (SCORE 2)	Visible deformation above ankle (SCORE 3)	Visual deformation above knee (SCORE 4)
Pleural effusion	No pleural effusion (SCORE 0)	Minor (non-amendable for puncture of pleural effusion) (SCORE 2)		Major (amendable for puncture of pleural effusion) (SCORE 3)	
Ascites	No ascites (SCORE 0)	Minor ascites, only detected by echocardiography (SCORE 2)		Significant ascites (SCORE 3)	

ADVOR congestion score: composite clinical congestion score used in the ADVOR trial to quantify signs of volume overload, based on the degree of peripheral oedema, pleural effusion, and ascites.

The study is approved by local institutional ethics committees and conducted in accordance with the Declaration of Helsinki and the International Conference on Harmonization Good Clinical Practice guidelines. All participants will provide written informed consent. The study is registered at ClinicalTrials.gov (NCT06285760).

### Eligibility criteria

Inclusion and exclusion criteria are summarized in *Table 2*. Eligible patients will be those admitted with AHF who present at least one clinical sign of volume overload (peripheral oedema, ascites, or pleural effusion) and elevated natriuretic peptides [N-terminal pro-B-type natriuretic peptide (NT-proBNP) >1000 pg/mL or brain natriuretic peptide (BNP) >250 ng/mL]. Patients must have been treated with intravenous furosemide and an SGLT2i for the previous 24 h and still have residual congestion (ADVOR score >1).

**Table 2:** Eligibility criteria

Inclusion criteria
Admission with a clinical diagnosis of HF and ≥1 clinical sign of volume overload (i.e. oedema, ascites or pleural effusion) Plasma NT-proBNP level >1000 pg/ml or BNP level >250 ng/ml at admission. Treatment with furosemide according to European guidelines and an SGLT2i, with an indication for a second diuretic due to residual congestion (defined as ADVOR score >1).

Exclusion criteria
Systolic blood pressure <90 mmHg or mean arterial pressure <65 mmHg. Expected use of intravenous inotropes, vasopressors or sodium nitroprusside at any time point during the admission Contraindication to SGLT2i Type 1 diabetes Estimated glomerular filtration rate <20 ml/min/1.73 m^2^ or use of renal replacement therapy or ultrafiltration at any time before study inclusion Exposure to nephrotoxic therapy, as contrast dye, anticipated during the admission Congenital heart disease requiring surgical correction Maintenance treatment with acetazolamide Inability to accurately measure urine output. Serum potassium <3,5 mEq/L Venous pH <7,30 Concurrent diagnosis of an acute coronary syndrome Severe aortic stenosis or obstructive hypertrophic cardiomyopathy Cardiac transplantation or ventricular assist device Subjects who are pregnant or breastfeeding History of sulphonamide allergy, liver cirrhosis or nephrolithiasis.

HF, Heart Failure; NT-proBNP, N-terminal pro-B-type natriuretic peptide; BNP, B-type natriuretic peptide; SGLT2i, sodium–glucose cotransporter-2 inhibitors

Key exclusion criteria will include systolic blood pressure <90 mm of mercury (mmHg) or mean arterial pressure <65 mmHg, maintenance therapy with acetazolamide, contraindications to SGLT2i, estimated glomerular filtration rate <20 ml per minute per 1.73 square meters (mL/min/1.73 m^2^) or prior renal replacement therapy, anticipated exposure to nephrotoxic contrast agents, acute coronary syndrome, serum potassium <3.5 milliequivalents per litre (mEq/L), or venous pH <7.30.

### Objectives and endpoints

#### Primary endpoint

The primary endpoint will be the change in natriuresis, measured in millimoles per litre (mmol/L) from baseline (24-h pre-acetazolamide) to 24 h after initiation of acetazolamide, assessed using 24-h urine collections.

#### Secondary endpoints

Secondary endpoints will include changes in the following parameters from baseline (24-h pre-acetazolamide) to 24 and 48 h after initiation of acetazolamide:

- Diuresis (litres)
- Body weight (kg)
- Clinical congestion assessed by ADVOR score. The ADVOR congestion score is a 0–10 scale used in the ADVOR trial^8^ to objectively quantify volume overload in AHF, based on three components: peripheral oedema scored from 0 to 4 points, pleural effusion from 0 to 3 points, and ascites from 0 to 3 points. A total score of 0 represents the absence of measurable congestion, while the maximum of 10 indicates severe, multi-site fluid accumulation.
- Ultrasound parameters of congestion, including inferior vena cava (IVC) diameter, Venous Excess Ultrasound (VExUS) score, and lung B-lines. The VExUS score is an ultrasound-based grading system used to assess systemic venous congestion by integrating measurements of the IVC and Doppler patterns of key abdominal veins, including the hepatic vein, portal vein, and intrarenal veins. A dilated IVC combined with progressively abnormal venous Doppler waveforms indicates increasing venous congestion. VExUS is typically graded from 0 to 3, where Grade 0 represents no significant congestion and Grade 3 indicates severe venous congestion with markedly abnormal waveforms across multiple veins. The lung B-lines will be assessed by the 8-zone lung ultrasound method, that evaluates pulmonary congestion by scanning four zones on each haemithorax (anterior and lateral, each divided into upper and lower fields). In each zone, the operator assesses the pleural line and counts B-lines. A zone is considered positive if it contains more than three B-lines.

#### Safety endpoints

Safety endpoints will include changes in renal function assessed by serum creatinine, serum electrolytes, bicarbonate levels, and systolic blood pressure within 24 and 48 h following acetazolamide administration. Worsening renal function is defined as an increase in serum creatinine of >0.3 milligrams per decilitre (mg/dL) relative to baseline. Clinically relevant hypotension is defined as symptomatic hypotension with a systolic blood pressure <100 mmHg. Metabolic acidosis is defined as a pH <7.2 and serum bicarbonate <20 mmol/L.

### Study procedures

The study procedures are summarized in the *Central Illustration*.

To minimize inter-centre variability, a comprehensive investigator’s manual was developed and distributed to all participating sites prior to study initiation. This document provided detailed methodological guidance for interpreting VExUS and standardized instructions for calculating the ADVOR congestion score. In addition, dedicated training sessions were conducted to familiarize investigators with the study protocol and ensure consistent application of the assessment methods across centres. All evaluations were performed by physicians with specific expertise in heart failure, further contributing to the standardization and reliability of measurements across participating sites.

### Visit 0: admission for acute heart failure

Upon admission for AHF with clinical signs of congestion, the standard loop-diuretic strategy recommended by the European guidelines will be implemented.^19^ An initial intravenous dose of furosemide equivalent to 1–2 times the patient’s chronic oral dose will be given. Patients not previously treated with loop diuretics will receive a 20–40 mg intravenous bolus.

Diuretic response will be evaluated within 2–6 h by measuring urine output and urinary sodium in a spot urine sample. In cases of poor diuretic response, the furosemide dose will be doubled and reassessed, up to a maximum daily dose of 400 mg.

#### Visit 1: initiation of SGLT2i and baseline assessment

Within the first 24 h of admission, eligible patients who provide written informed consent will be enrolled. At that time, an SGLT2i will be initiated if not previously prescribed.

During this visit, medical history, physical examination, laboratory parameters, concomitant treatments, and ultrasound findings will be recorded. A 24-h urine collection will be started to quantify natriuresis and urinary electrolyte excretion.

#### Congestion assessment

A multiparametric assessment of congestion will be performed by the treating physician at the time of the study inclusion and daily thereafter.

This evaluation will include the ADVOR score, comprising peripheral oedema, pleural effusion, and ascites, as well as ultrasound parameters such as IVC diameter, pleural effusion, and pulmonary B-lines on lung ultrasound.

### Visit 2: first acetazolamide dose

If congestion (defined as an ADVOR score >1) persists after 24 h of treatment with intravenous furosemide and an SGLT2i, intravenous acetazolamide (500 mg) will be administered.

Before acetazolamide therapy, clinical evaluation, laboratory testing, and ultrasound assessment will be repeated. A second 24-h urine collection will be initiated to assess natriuresis and urinary electrolyte excretion.

#### Preparation and administration of treatment

Each 500 mg vial of acetazolamide will be reconstituted with 5 mL sterile water for injection and administered as an intravenous bolus with 10 mL of 0.9% saline.

In addition, a continuous infusion of 5% dextrose containing 3 g of magnesium sulphate will be initiated.

#### Visits 3 and 4: 24 and 48 h after acetazolamide

If congestion persists at Visit 3 (ADVOR score >1), a second 500 mg dose of acetazolamide will be given, and the magnesium sulphate infusion will be continued. A final 24-h urine collection will be performed.

#### Visit 5: hospital discharge

At discharge, a comprehensive evaluation will be conducted using the same methodology as Visit 1, including physical examination, ultrasound assessment, laboratory testing, and documentation of prescribed medical therapy. Potential adverse events and in-hospital complications will also be reviewed and recorded.

#### Visits 6 and 7: 30-day and 90-day follow-up

At 30 and 90 days following acetazolamide therapy, patients will undergo clinical and ultrasound evaluations according to the same standardized protocol. Concomitant medications and adverse events, including heart failure readmissions and mortality, will be documented.

### Statistical plan

#### Sample size and power calculator

In the ADVOR study, a 20% increase in natriuresis was observed in the group of patients treated with acetazolamide. Therefore, considering an alpha error of 5% and a power of 80%, the approximate sample size to detect a 20% increase in natriuresis after adding acetazolamide to standard treatment is 64 patients. No adjustment for dropouts is applied, as the primary endpoint is assessed during a short, closely monitored inpatient period in which loss to follow-up is expected to be minimal.

#### Statistical analysis

Categorical variables will be summarized as absolute and relative frequencies, and continuous variables as mean (standard deviation) or median (interquartile range), depending on the normality assumption. The analysis for the primary endpoint will be developed through the pre-post comparison using paired *t*-test or Wilcoxon rank-sum test for paired samples. For the secondary outcomes, longitudinal random-effects models will be fitted to evaluate changes in urinary sodium levels as well as other outcomes across study visits. Using visit 1 (pre-acetazolamide) as the reference category, the model will account for both within-individual and between-individual variability, assuming no correlation between the random intercepts and the covariates. Adjusted analyses for the time from SGLT2i initiation, specific SGLT2i used, and loop diuretic dose will be done. The predicted values will be estimated with the corresponding 95% confidence intervals. Significance level will be set at 0.05 and Stata v18 will be the software used to perform the analysis.

Missing data will be handled according to the nature of each endpoint. For the primary endpoint, patients with incomplete 24-h urine collections will be excluded from the primary analysis. Secondary endpoints will be analysed using available data. Given the short and closely monitored inpatient period during which the primary endpoint is assessed, missing data are expected to be minimal. For follow-up assessments at 30 and 90 days, analyses will be performed using available cases without imputation.

## Discussion

In the SANDI study, we will prospectively evaluate whether the addition of intravenous acetazolamide to guideline-directed therapy with intravenous loop diuretics and SGLT2i enhances natriuresis and diuresis in patients hospitalized for AHF with persistent congestion. Given the study design, the aim is not to establish causality but to describe clinical changes and the impact on natriuresis following the introduction of acetazolamide. To our knowledge, this will be the first study specifically designed to assess the incremental benefit of acetazolamide in patients already treated with SGLT2i.

### Combined diuretic therapy in congestive acute heart failure: still gaps in the evidence

One of the primary therapeutic goals in congestive AHF is effective decongestion. Current recommendations for initial intravenous loop diuretic therapy are primarily derived from the Diuretic Optimization Strategy Evaluation (DOSE) trial,^25^ published more than 10 years ago. Although most patients with AHF respond adequately within the first hours of treatment, a significant proportion remain with fluid overload, which is associated with prolonged hospitalization and worse outcomes.^26,27^

While robust clinical evidence defining the optimal sequencing of diuretic therapy is lacking, growing data support the early combination of agents with complementary mechanisms to achieve euvolaemia.^7,8^ It should be noted, however, that at the time these studies were conducted, SGLT2i were not yet considered part of guideline-directed medical therapy for heart failure. The Acute Heart Failure with Volume Overload Guided by Serial Spot Urine Sodium Assessment (DECONGEST; NCT05411991) study will evaluate the efficacy of combining acetazolamide with chlorthalidone, a diuretic with more established natriuretic efficacy in AHF. Nevertheless, the objective of co-administering SLGT2i and acetazolamide in this study is rather to address the combined effect of proximal tubular sodium handling induced by SGLT2 inhibition and acetazolamide in the contemporary era, where SGLT2i are increasingly used in patients with heart failure across the entire spectrum of ejection fraction.

### Sodium–glucose cotransporter-2 inhibitors in acute heart failure

SGLT2i therapy has an established prognostic role in chronic heart failure and may confer clinical improvements when initiated during hospitalization. The benefits of SGLT2i in heart failure extend well beyond their diuretic effects,^9^ although diuresis represents one of their contributing mechanisms of action: inhibition of glucose and sodium reabsorption in the proximal tubule, leading to osmotic diuresis driven by glycosuria. SGLT2i also modulate NHE3 activity, contributing to modest natriuresis, though compensatory distal tubular mechanisms may attenuate this effect.^17,19^

Several trials have evaluated early SGLT2i therapy in AHF in combination with loop diuretics, aiming to enhance decongestive efficacy and improve long-term outcomes.^12–15^ To date, studies have shown heterogeneous results regarding their impact on diuresis, natriuresis, and overall decongestion. While most trials reported increased urine output and reductions in natriuretic peptides, findings on urinary sodium excretion have not been consistent.

Notably, in a substudy from the EMPULSE trial,^28^ the effects of empagliflozin on five prespecified markers of congestion were analysed, and the relationship between effective decongestion and 90-day clinical benefit was assessed. Compared with placebo, patients receiving empagliflozin showed significantly greater reductions in all decongestive parameters at all assessed time points (days 15, 30, and 90). Moreover, patients with greater weight loss were more likely to experience a net clinical benefit at 90 days.

### Acetazolamide as an adjunct to loop diuretics in AHF

The natriuretic effect of acetazolamide results from inhibition of carbonic anhydrase in the proximal tubule, which prevents the conversion of carbon dioxide and water into bicarbonate and hydrogen ions, thereby interfering with the NHE3.^29^ As monotherapy, its diuretic potency is limited, producing a fractional sodium excretion of approximately 4%.^20^ However, when combined with loop diuretics, its efficacy is markedly increased.^8,30–33^

The DIURESIS-CHF trial investigated intravenous acetazolamide in combination with loop diuretics versus high-dose loop diuretics alone.^34^ No significant differences were found in urinary sodium, NT-proBNP, or clinical decongestion. However, recruitment was incomplete, leading to underpowering. More recently, the ADVOR trial demonstrated that adding intravenous acetazolamide to loop diuretics significantly improved decongestion and natriuresis, and shortened hospital stay, underscoring the role of acetazolamide as an effective adjunctive diuretic in the management of AHF.^8^

Notably, none of these studies included patients already treated with SGLT2i, which, like acetazolamide, primarily exert their diuretic effects by modulating sodium handling in the proximal segment of the nephron.

### Acetazolamide and SGLT2i: potential synergy in the proximal nephron

The proximal nephron reabsorbs approximately 60–70% of filtered sodium, primarily via NHE3. In heart failure, NHE3 is upregulated, promoting sodium and water retention.^16^ Inhibition of proximal tubular sodium reabsorption can therefore potentiate the natriuretic response to loop diuretics.

SGLT2i and acetazolamide both target proximal sodium handling but through distinct, partly overlapping mechanisms. SGLT2i alter glucose–sodium transport dynamics, indirectly reducing NHE3 activity, while acetazolamide directly inhibits carbonic anhydrase, decreasing bicarbonate reabsorption and further suppressing NHE3.^16,18^ Consequently, their clinical effects differ: SGLT2i produce modest natriuresis with sustained metabolic and cardiovascular benefits, whereas acetazolamide exerts a more pronounced natriuretic effect suitable for short-term intensification of diuretic therapy in acute settings.

The potential additive or synergistic effects of these agents remain unexplored. Ongoing trials—NCT06535529, NCT06783166, and *Study on Acetazolamide with Empagliflozin and Dapagliflozin for Patients with Acute Heart Failure and Fluid Retention*—are crucial for determining optimal strategies for proximal tubular diuretic modulation and defining the future role of combined acetazolamide–SGLT2i therapy in acute decompensated heart failure.

### Clinical implications

The results of this mechanistic study will provide insight into the clinical relevance of targeting the proximal tubule in patients treated with both loop diuretics and SGLT2i. Whether acetazolamide retains additive natriuretic efficacy in the setting of ongoing SGLT2 inhibition is a key unanswered question with direct clinical relevance. By quantifying changes in urinary sodium excretion and diuretic response before and after acetazolamide administration, this study aims to clarify the potential value of dual proximal tubule modulation. Identifying effective strategies to optimize natriuresis is crucial, as poor natriuretic response is associated with worse clinical outcomes. The findings of SANDI may therefore help inform future algorithms for diuretic sequencing in contemporary clinical practice.

### Limitations

This study has several limitations. First, this is an observational, non-randomized study; therefore, causal inferences will be limited despite the within-subject design, which minimizes inter-individual variability. Therefore, the findings should be taken as associative and hypothesis-generating. Second, although the sample size is appropriate for detecting changes in natriuresis, it may be insufficient to assess clinical outcomes or infrequent adverse events. Third, the ADVOR congestion score and ultrasound measures are not conducted in a blinded manner, which may introduce potential observer bias in the evaluation of congestion. In addition, its multicentre design may introduce measurement variability across participating sites, particularly for assessments such as the ADVOR congestion score and the VExUS evaluation, which may be subject to operator-dependent interpretation. Although efforts were made to standardize these assessments through a detailed investigator’s manual and dedicated training sessions prior to study initiation, some residual inter-centre variability cannot be completely excluded. Fourth, natriuresis will serve as the primary surrogate endpoint; although mechanistically and prognostically relevant, it may be influenced by dietary intake, renal function, and neurohormonal tone. Fifth, while treatment will follow guideline-directed care, some variability in supportive therapy may occur, reflecting real-world clinical practice. Finally, the sample size estimation was based on the effect size observed in the ADVOR trial, which reported an approximately 20% increase in natriuresis following acetazolamide administration. However, potential variability in natriuretic response related to concomitant SGLT2 inhibitor therapy has not been fully characterized in AHF populations and could influence the magnitude of the observed effect. Nevertheless, the within-subject pre–post design, in which each patient serves as their own control, may reduce inter-individual variability and improve the ability to detect changes in natriuresis in this mechanistic, hypothesis-generating study.

## Conclusions

The SANDI study will address an important evidence gap by evaluating whether adding intravenous acetazolamide to intravenous loop diuretics and SGLT2i enhances natriuresis and diuresis in patients with AHF and persistent congestion. These results will help define the role of proximal-acting diuretic combinations in modern heart failure management.

## Declarations

### Data Availability

No data were generated or analysed for this manuscript.
